# Confirming Silent Translocation through Nanopores with Simultaneous Single-Molecule Fluorescence and Single-Channel Electrical Recordings

**DOI:** 10.1021/acs.analchem.3c02329

**Published:** 2023-11-22

**Authors:** Daniel L. Burden, Joshua J. Meyer, Richard D. Michael, Sophie C. Anderson, Hannah M. Burden, Sophia M. Peña, Kristin Joy Leong-Fern, Lily Anne Van Ye, Elizabeth C. Meyer, Lisa M. Keranen-Burden

**Affiliations:** Chemistry Department, Wheaton College, Wheaton, Illinois 60187, United States

## Abstract

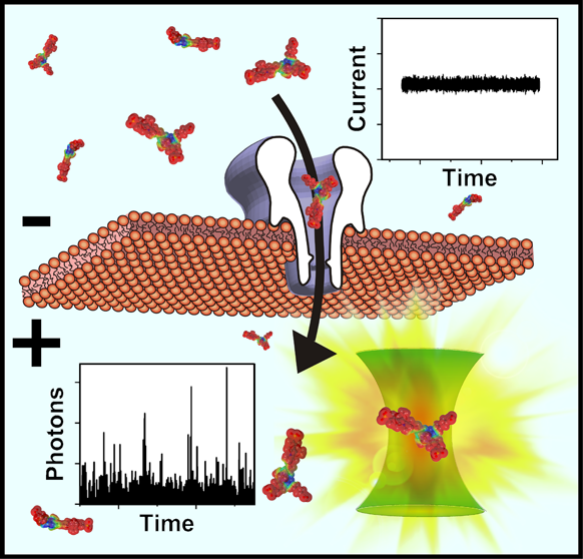

Most of what is known concerning the luminal passage of materials through nanopores arises from electrical measurements. Whether nanopores are biological, solid-state, synthetic, hybrid, glass-capillary-based, or protein ion channels in cells and tissues, characteristic signatures embedded in the flow of ionic current are foundational to understanding functional behavior. In contrast, this work describes passage through a nanopore that occurs *without* producing an electrical signature. We refer to the phenomenon as “silent translocation.” By definition, silent translocations are invisible to the standard tools of electrophysiology and fundamentally require a simultaneous ancillary measurement technique for positive identification. As a result, this phenomenon has been largely unexplored in the literature. Here, we report on a derivative of Cyanine 5 (sCy5a) that passes through the α-hemolysin (αHL) nanopore silently. Simultaneously acquired single-molecule fluorescence and single-channel electrical recordings from bilayers formed over a closed microcavity demonstrate that translocation does indeed take place, albeit infrequently. We report observations of silent translocation as a function of time, dye concentration, and nanopore population in the bilayer. Lastly, measurement of the translocation rate as a function of applied potential permits estimation of an effective energy barrier for transport through the pore as well as the effective charge on the dye, all in the absence of an information-containing electrical signature.

Single-molecule electrical signals generated in biological,^1^ solid-state,^2^ synthetic,^3^ hybrid,^4^ and capillary-based nanopore systems,^5^ as well as ion-channel recordings in cells and tissues,^6^ generally arise from two underlying interaction types. The first involves movement of a target compound through the pore. During transit, the target excludes ions moving through the lumen, or it momentarily changes the electric field within the pore, to produce a brief reduction in ion current.^7−9^ Electrical blockage arising from translocation allows evaluation of size,^5^ shape,^10,11^ charge,^12^ binding capacity,^13^ conformational change,^14^ concentration,^15^ or other important characteristics relevant to the transported species or the nanopore. The second category involves molecules that do not pass through the pore.^16^ In this case, electrical signals arise from temporary specific, or nonspecific, binding of compounds to the inner or outer surface of the pore.^17,18^ In some instances, compounds might move well inside the pore but do not fully traverse the nanopore interior.^19,20^ These momentary interactions produce a corresponding alteration of ion flow, by direct obstruction, by inducing a conformational change in the pore, or by generally altering the electric field.^21^ Nontranslocating electrical blockages result in fluctuating current signatures that can also be used to characterize the interacting compound or the pore. Historically, electrical events from both translocating and nontranslocating interaction types have enabled the detection of many different chemical species, including ions,^22,23^ small molecules,^24^ and a wide range of polymers.^25−28^

However, a third type languishes in relative obscurity due to present limits in metrology. This category includes compounds that translocate through the pore lumen but do not produce a detectable electrical signature or any kind of measurable pattern that can be distinguished from background noise. Such compounds are electrically silent. Notably, silent translocations have been recognized in the context of fast-moving polymer strands of nucleic or amino acids.^29,30^ When an extended molecular chain passes through a nanopore too quickly, individual monomers within the strand can be hidden from characterization, making the translocation of these nucleotides effectively silent. Indeed, reducing translocation velocity to maximize the extractable information content has been a significant challenge in the advancement of nanopore-based DNA and protein sequencing.^31,32^ As a general category, however, silent translocations encompass a much wider array of compounds. Electrical quiescence for any target molecule, ion, macromolecule, polymer, or particle is possible and even quite probable, especially if the translocator is small in comparison to the dimensions of the pore lumen. Silent molecular translocations most likely occur in a wide variety of nanopore systems. But the literature is nearly void of their discussion because positive evidence of occurrence is difficult to produce and because the phenomenon has never been intentionally sought or studied in detail.

Yet, silent molecular translocations might have profound implications for fundamental biology, physiology, neuroscience, and biophysical science as well as pragmatic impact for the future development of sensors. Cellular signals frequently arise from a cascade of reactions initiated by one or a small number of molecules that interact with membrane channels to propagate nerve impulses,^33^ stimulate muscle activity,^34^ or assist with the delivery of proteins.^35^ The ability to explore the silent activity of transportable signaling compounds could provide new insights into existing, or yet-to-be-discovered signal-transduction mechanisms in cells^6^ or protocells.^36^ Additionally, a more detailed understanding of the types of molecules that undergo silent translocation in a given nanopore sensing system could guide design principles to improve the future performance of nanopore-based analysis, especially in complex chemical matrices.^37^ Or, detecting silent translocations might further enable single-molecule-level separations that occur in aggregate nanopore structures, such as the nuclear pore complex and its synthetic mimics.^38−41^ Likewise, a more robust understanding of the specific principles that create silent translocation might eventually generate theories to predict what compounds will be electrically detectable and what compounds will not.

While the electrical capability of nanopore measurement systems is constantly improving to reveal more interaction details with an ever-increasing variety of species,^42^ the likely prevalence of silent translocators motivates the development of new fluorescent measurement techniques that possess acute sensitivity and high time resolution. This advance would permit direct confirmation, or refutation, of silent translocations in a host of nanopores and ion-channel systems by generating single-molecule^4^ electrical recordings directly alongside an optical method of detection.

Here, we employ highly sensitive single-molecule confocal fluorescence and single-channel electrical recordings simultaneously to explore the translocation of disulfo-cyanine 5 carboxylic acid (sCy5a) through individual α-hemolysin (αHL) nanopores. The nanopores are embedded in a lipid bilayer that is suspended over an aqueous-filled microwell. A number of techniques have been demonstrated for optically monitoring the movement of an ensemble of dye molecules, or ions that bind to dye molecules, through individual nanopores.^44−47^ Simultaneous electrical and fluorescence recordings of nanopipette transport^48^ and fluorescently labeled ion channels in bilayers^49−52^ have also been published. Our latest and less experimentally demanding approach employs a device for trapping trace quantities of translocated material in a closed aqueous-filled microcavity. Microelectrodes deposited within the cavity interrogate free-standing lipid bilayers formed by traditional painting techniques.^53^ We probed the aqueous microwell using a confocal fluorescence microscope. The optics of the microscope are aligned to enhance the probability of detecting individual translocated dye molecules multiple times prior to photobleaching. Repeated molecular detection against a low background promotes rapid and exquisitely sensitive quantification of silently translocated molecules, even when the translocation rate is very low. Within the performance limits of standard electrical instruments, our findings confirm the occurrence of this inferred, but essentially uncharacterized phenomenon.

## Experimental Methods

Simultaneous single-molecule fluorescence and single-molecule electrical recordings were performed in a quadrature Microelectrode Cavity Array (MECAopto-inv, Nanion, Inc.). The basic chip characteristics and performance while probing the lipid bilayer with ensemble-level fluorescence techniques have been recently published.^54^ Each 150 μm microcavity contained a Ag/AgCl electrode ring and was constructed on top of a transparent coverglass, which allowed for optical access from an inverted microscope. Electrical recordings were also performed on MECA4 chips without an optical window. Lipid bilayers were painted over the top of the microcavities using diphytanoyl phosphatidyl choline (DPhPC, Avanti Polar Lipids) in octane (Sigma-Aldrich), which formed a partition between a small aqueous-filled compartment below the membrane and the large main fluid-filled well of the chip. The main well also contained a Ag/AgCl electrode that permitted the application of a transmembrane voltage and the collection of low-noise current recordings. Electrical recordings were acquired using the built-in four-channel amplifier of an OrbitMini (Nanion, Inc.). Wild-type αHL was acquired from Sigma-Aldrich and was also produced in-house using procedures published previously.^55^ The recording media (either 1 M KCl, 1 M NaCl, or 1 M LiCl, Sigma) was buffered at pH 7.5 in 10 mM Tris-HCl (EMD Millipore Corp.).

Single-molecule fluorescence recordings were performed on a Nikon Ti-E inverted optical microscope that was custom-adapted for confocal operation. A Nikon 100×, Plan Apo TIRF, N.A. 1.49 objective was used to create the confocal detection volume, which was positioned inside the bilayer-capped microwell of the MECAopto-inv. Fluorescence excitation/emission of sulfo-cyanine 5 carboxylic acid (sCy5a, BroadPharm) was performed at 640 and 655–705 nm, respectively. Fluorescence photons from single molecules were detected using a low-dark-count avalanche photodiode (SPCM-AQHR, Excelitas). Output from the photodetector was recorded using a multichannel scalar set to record the photon stream continuously in 1 ms time bins. Synchronization of the optical and electrical recordings was accomplished by using custom-written software. See the Supporting Information for further details.

## Results and Discussion

**Figure 1:**
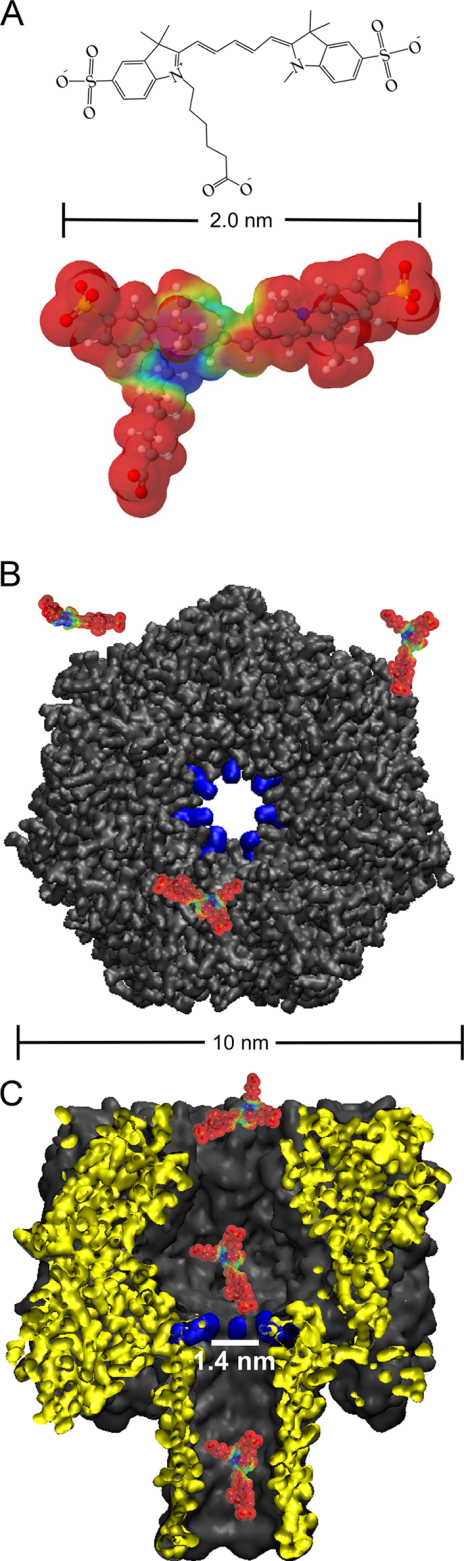
Structure and size of disulfo-cyanine 5 carboxylic acid (sCy5a) and the contour of the αHL lumen. (A) sCy5a possesses conformational flexibility around single bonds that likely assist with full translocation. A molecular electrostatic potential map (red = negative, blue = positive) of the van der Waals surface illustrates the variable charge distribution over the structure, which has a net charge of −2. (B) αHL allows entry of sCy5a from the cap (*cis*) side with multiple dye orientations. (C) Passage beyond the barrier arising from the ∼1.4 nm constriction site (blue, LYS147) is less probable and most likely requires specific sCy5a orientation.

A comparison of sCy5a geometry (Figure 1A) to the interior cross section of αHL (Figure 1B) suggests that movement of sCy5a beyond the narrowest constriction site is possible but perhaps not favorable. Penetration from bulk solution into the *cis*-side vestibule most likely occurs with a high probability, accommodating multiple orientations of sCy5a upon entry. However, full translocation can only take place if sCy5a encounters the constriction region (Figure 1B,C, blue) with the proper orientation. We presume incursion beyond the narrow constriction site (∼1.4 nm diameter) results in irreversible transport through the β-barrel and into the compartment on the *trans* side of the nanopore. Given the net charge of −2 on sCy5a (Figure 1A, negative surface potential in red, positive regions in blue), the application of a positive transmembrane potential (*cis* chamber grounded) enhances the probability of full translocation due to the generation of directional electrophoretic and electroosmotic flow. These forces work to draw sCy5a from the *cis*-side vestibule, past the constriction site, and through the β barrel. Published calculations indicate that, for the relatively small potentials utilized in this study (−50 to +150 mV), electrophoretic and electroosmotic flow begin to outcompete Brownian motion only at distances very near the constriction.^43^ Thus, entry of sCy5a into, and movement within, the αHL vestibule is dominated by random diffusive motion. In fact, a significant fraction of molecules that enter the vestibule escape and return to regions outside the nanopore.^43^ However, upon encountering the constriction site, the probability of full translocation is enhanced by the limited conformational flexibility of sCy5a, in both the conjugated backbone connecting the aromatic rings and the nonconjugated carboxylic acid chain. Given the overall size and negative charge of sCy5a, along with the 7 positively charged residues comprising the constriction region (LYS147), it is noteworthy that a drop in ionic flow through the nanopore cannot be detected.

**Figure 2:**
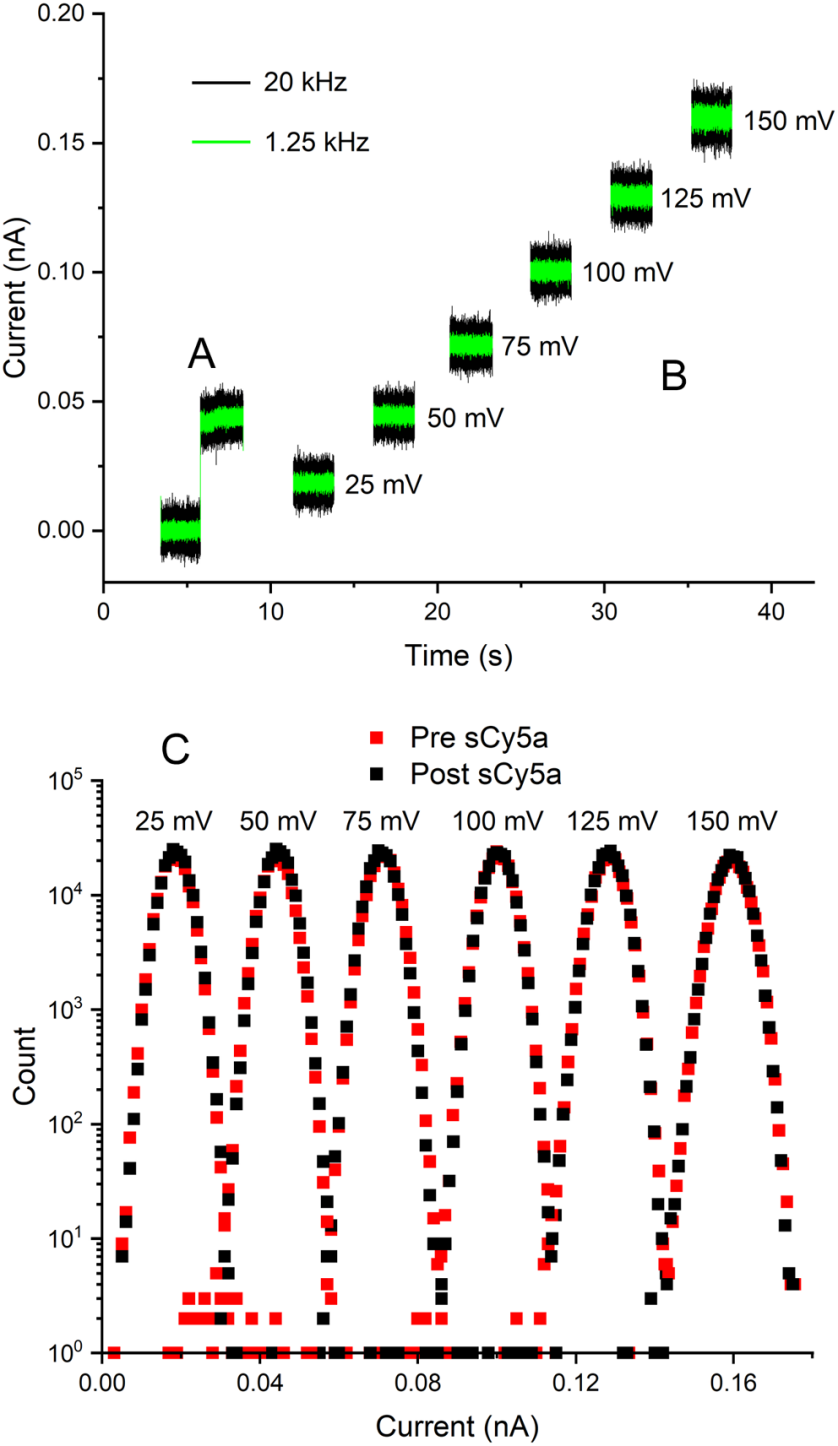
Single-channel current recordings provide no evidence of sCy5a translocation. (A) Single-channel insertion at 50 mV. (B) Representative current recordings at various potentials (filtered at 20 and 1.25 kHz) after the addition of 3 μM sCy5a. No translocation signatures are apparent in either recording over a range of potentials. (C) All-points current histograms (sampled at 20 kHz for 10 s) from each potential in (B) before (red) and after (black) sCy5a addition. Peak overlap underscores the lack of current fluctuation induced by sCy5a. Data shown were collected in 1 M KCl, but they are typical of all conditions tested. Current recordings in 1 M NaCl and 1 M LiCl also remain quiescent without the appearance of blockage events or detectable noise fluctuations.

Figure 2 shows data collected under a variety of measurement conditions with no evidence of added noise or other electrical signatures. We define an electrical event as a deflection in current that is at least 3 standard deviations beyond the average background noise level. Detection events also have to occur frequently enough to be distinguished (with 99.9% confidence) from other rare events caused by the presence of solution contaminants or infrequent nanopore gating events that arise from random conformational fluctuation. Within the range of conditions that we explored, no statistically distinguishable signatures appear. Apparently, the electrostatic attraction between the positive ring of lysine at position 147 and sCy5a is insufficient to produce a statistically significant change in the ionic flow through the pore. The lack of current alteration suggests that sCy5a slips through the constriction rapidly or that the structural profile of the nanopore prohibits passage of sCy5a altogether. These two opposing possibilities underscore the necessity of performing simultaneous orthogonal measurements at the fundamental limits of detection sensitivity to critically test if translocation occurs. Interestingly, our combined single-molecule electrical and single-molecule optical observations indicate that sCy5a does indeed translocate through αHL. However, surprisingly, it does so with electrical silence.

We stress, however, that electrical silence can be defined only up to the noise-bandwidth limitations of the electrical recording apparatus. Given a system with no noise, no stray capacitance, and essentially infinite bandwidth, it seems unlikely that sCy5a, with its relative size and electrostatic attraction to the residues of the constriction, would fail to register an electrical signal. Furthermore, the proper combination of salts, solution additives, and pore modification should create conditions that are more favorable for electrical detection.

To test this idea, we explored a set of salts (e.g., KCl, NaCl, LiCl) which have been shown to slow DNA translocation through a variety of nanopores, including αHL, by up to a factor of 10.^31^ Nonetheless, we did not observe electrical evidence for sCy5a passage, rendering it silent under all of the conditions we examined. It is worth noting that “absolute” electrical silence under all possible measurement conditions can never be experimentally verified. This inability does not, however, lessen the pragmatic value associated with the capacity to confirm or refute the passage of silent translocators using simultaneous measurement schemes for any given nanopore under any particular set of measurement conditions.

**Figure 3:**
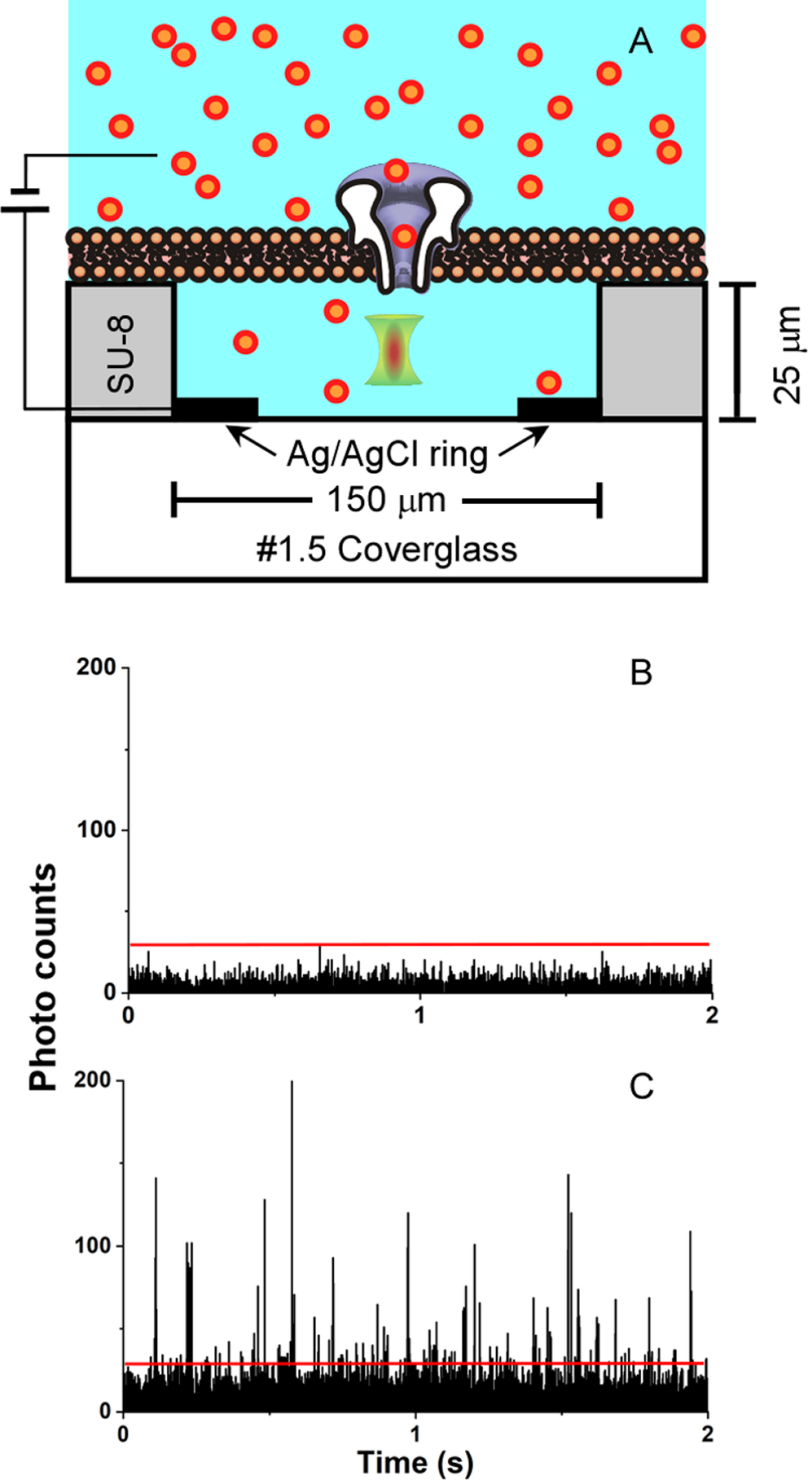
Optical detection using a MECAopto-inv chip with a Ag/AgCl microelectrode deposited at the bottom of a 440 pL microcavity fashioned in an SU-8 polymer layer (3A). Fluorescence emission from sCy5a (655–720 nm) is excited at 640 nm. Prior to dye accumulation in the cavity, the bilayer-capped microwell is photobleached to reduce background count levels (3B). Delivery of sCy5a to the top chamber and application of +100 mV to the membrane results in photobursts appearing in the microwell (3C). sCy5a produces a photoburst each time a molecule diffuses into the stationary optical probe, which is counted when the photon flux exceeds a threshold (red line). The 2 s optical recording displayed in 3C was acquired after applying +100 mV for 30 s with 3 μM sCy5a located above a membrane containing 28 open nanopores.

Positive evidence for silent translocation arises from single-molecule confocal fluorescence microscopy performed in an aqueous microwell (Figure 3A). Figure 3B,C shows a typical single-molecule fluorescence recording before and after the application of a positive potential that drives sCy5a through the pore. Photon bursts from the 440 pL microcavity occur each time a sCy5a molecule enters into or crosses the optical detection volume, which is positioned near the middle of the microcavity. Comparison of panels B and C reveals a dramatic increase in the single-molecule fluorescence burst rate following a period of applied potential. Counting the number of burst events that exceeds a background-discriminating threshold permits the measurement of concentration and the total number of translocated dye molecules. An electrical current recording, captured simultaneously across the bilayer that caps the microwell, allows direct determination of the number of open αHL nanopores during the accumulation period. We performed numerous measurements with membranes containing 1–150 open αHL nanopores, a range of potentials (−50 to 150 mV), and various accumulation periods (1–425 s). After comparing to adequate control and blank optical recordings, including bilayers without any nanopores present (see the SI for details), we conclude these photon bursts do indeed result from sCy5a dye molecules that are delivered to the microcavity by passage through αHL. In all cases, the simultaneously acquired electrical recordings offer no evidence of sCy5a translocation through the pore. Together, these two observations provide powerful evidence that sCy5a is a silent translocator.

**Figure 4:**
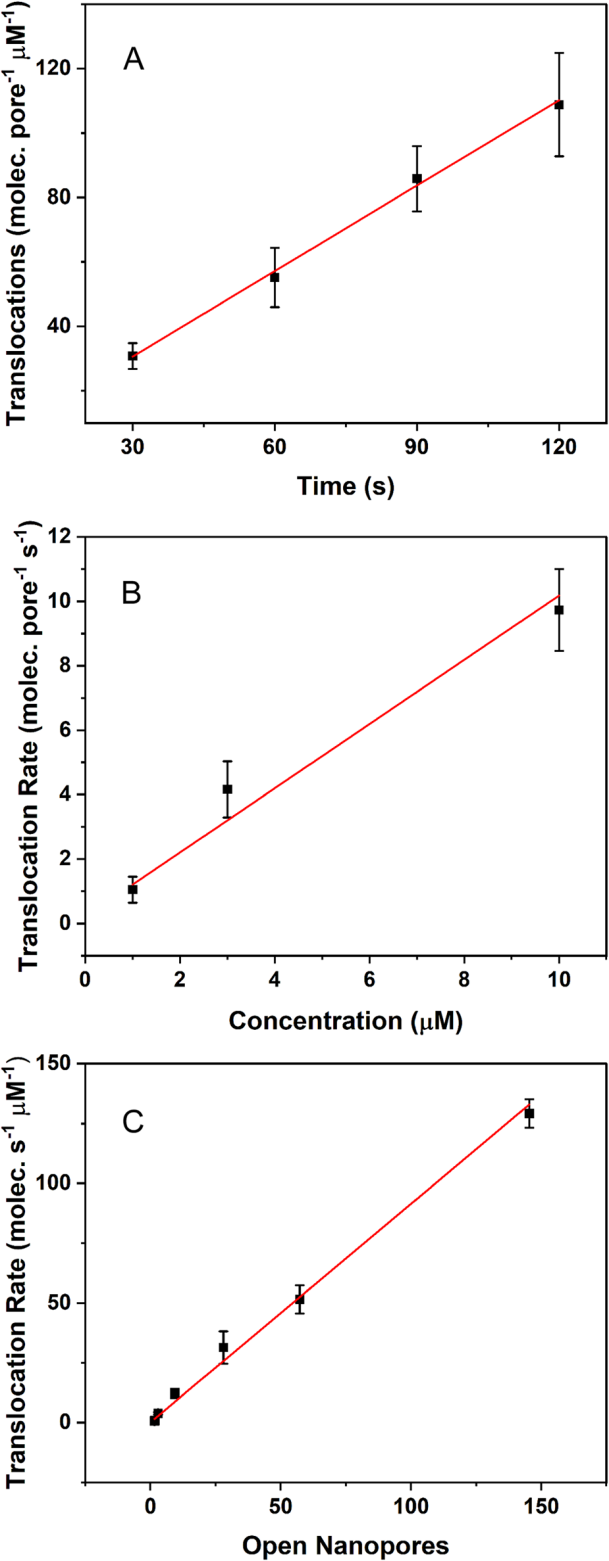
(A) Number of translocated sCy5a molecules increases linearly as a function of accumulation time (slope ∼0.90). (B) Translocation rate increases linearly as a function of sCy5a dye concentration located on the *cis*-side of the membrane (slope ∼1.0). (C) Translocation rate increases linearly with the number of open αHL nanopores in the membrane (slope ∼0.91). All measurements were performed at +100 mV applied potential. All data sets (A–C) were acquired with various numbers of open nanopores in the bilayer, dye concentrations, and accumulation times and are normalized for comparison. Error bars (±1 standard deviation) and averages were determined from *N* = 3 measurements.

Further positive evidence for sCy5a translocation arises from a change in the number of dye molecules amassed in the microcavity as a function of accumulation time, dye concentration above the membrane, and number of open nanopores in the membrane. Assuming that each nanopore behaves as an open conduit, monotonic changes as a function of each tested variable are expected. Indeed, Figure 4 reveals the anticipated trends, which are all consistent with sCy5a translocation. All of the measurements in Figure 4A–C were performed with an applied potential of +100 mV, which drives translocation through individual channels at a rate just under 1 sCy5a molecule per second per μM. As can be seen, the slope of each data set is linear, and the slopes are also near 1. These relationships suggest there is little interaction, or clustering, of the nanopores assembled in the membrane. The formation of static, or dynamic, aggregates would likely deplete the local concentration of dye in proximity to a cluster and thereby decrease the observed dye translocation rate in a nonlinear fashion. Thus, within experimental uncertainty, each open nanopore appears to behave independently.

Partitioning and translocation of sCy5a into and through the nanopore are governed by three fundamental forces: an electrophoretic force that acts directly on the charged molecule under an electric field gradient, an electroosmotic force that arises within proximity of the nanopore due to the flow of water through the lumen, and Brownian motion. Both electrophoretic and electroosmotic forces are unidirectional. However, molecular diffusion is directionally random due to the inherent thermal energy distribution of molecules in solution. These three dynamic processes result in a distribution of sCy5a energies that factor into our observations. Furthermore, the net direction of Brownian motion is dependent on the orientation of the concentration gradient across the membrane. For the scenario tested here, where sCy5a molecules reside almost exclusively on one side of the bilayer, the net flux of molecules into and through the nanopore occurs from the high-concentration side of the bilayer to the other (as depicted in Figure 3A).

The rate of concentration-gradient-driven transport can either be assisted or opposed by electrophoretic and electroosmotic forces, depending on the polarity of the applied voltage. Moreover, at both positive and negative applied potentials, the nanopore presents a permanent barrier to translocation that inhibits the passage of a significant fraction of sCy5a molecules. With the *trans* chamber positive, the orientation of both electrophoretic and electroosmotic forces with respect to the nanopore is in the same direction as the concentration gradient.^43,56^ Thus, these forces assist the diffusive transport of sCy5a into and through the pore. However, at negative potentials, the direction of electrophoretic and electroosmotic forces is reversed, and the rate of transport across the pore is reduced but not abolished. At zero applied potential, the only factor contributing to translocation is diffusion. With no applied potential, we observe a small but measurable number of translocating sCy5a molecules. At increasingly more negative potentials, the measured transport rate drops exponentially.

Passage of sCy5a through αHL involves a complex interplay of factors, including steric hindrance from the nanopore geometry, local electric fields arising from charged amino acid residues within the pore, possible conformational fluctuations that might occur within the protein structure of the nanopore, the net charge on sCy5a, the size of the solvation sphere associated with sCy5a, the conformational flexibility of the sCy5a molecule, and the molecule’s translational energy. These complexities imply that individual molecules most likely encounter the energy barrier created by the nanopore interior differently. We surmise that these differences depend on a specific combination of these factors for any given molecule. We previously investigated a number of these interactions as they pertain to the electric fields generated by charged residues inside the lumen and the passage for spherical molecules of different sizes.^43^ However, for simplicity, in this study, we empirically consider only the overall magnitude of the apparent energy barrier as a function of applied voltage, which affects most, but apparently not all, sCy5a molecules. The model also considers the effective charge of the dye.

The data suggest the existence of at least two sCy5a populations that encounter the nanopore barrier in different ways (i.e., weak and strong interactions). We postulate a minority population that is characterized by negligible interaction with the barrier arising from sCy5a molecules that transit through the critical nanopore constriction region with optimal orientation, spatial conformation, and translational energy. These molecules translocate in a relatively unimpeded and potential-independent fashion. In pictorial terms, unhindered translocation could arise from sCy5a molecules that approach the constriction region in an extended conformation with an orientation parallel to the luminal axis of the pore and sufficient translational energy so that it slips by the barrier region with little interaction. This kind of translocation occurs for a statistically small but measurable number of encounters. We postulate a second population associated with the vast majority of sCy5a molecules that interact with the αHL barrier strongly. These molecules can be envisioned as approaching the nanopore barrier with an orthogonal orientation component, a folded or bulky conformation, a low translational energy, or some combination thereof.

We model the transport rate of sCy5a through the nanopore with an Arrhenius-like energy barrier.^57,58^ Our approach separates the overall translocation rate, *R*_m_^±^, according to the polarity of the applied voltage. At positive potentials, the model is further divided into two parts to account for the minority subpopulation that is presumably unaffected by the energy barrier. Thus, the measured rate at positive potentials, *R*_m_^+^, is the sum of a constant potential-independent term, *R*_*i*_, and a translocation term, *R*_p_^+^, that changes as a function of potential. The measured rate at negative potential, *R*_m_^–^, only requires a potential-dependent term, *R*_p_^–^. Thus
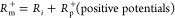
1
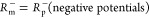
2

The translocation rate described by the potential-dependent terms, *R*_p_^±^, follows an Arrhenius transition-state relation, *kv* × e^–(*U*_eff_^±^–Δ*U*)/*kT*^, where *k* is a probability factor, *v* is a collisional frequency factor at the nanopore constriction, *U*_eff_^±^ denotes an effective barrier energy at either positive or negative potential, and Δ*U* accounts for the change in the barrier potential due to the applied voltage. The energy difference driving the charged dye through the pore is Δ*U =* |*z*_eff_|*eV*, where |*z*_eff_| is the size of the effective charge characterizing the dye, *V* is the transmembrane potential, and *e* is the magnitude of the elementary charge. Thus, eq 1 follows the general form of exponential growth with a constant offset.
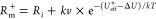
3

We note that in the limit of vanishing applied potential (i.e., Δ*U* ≈ 0), both *R*_p_^+^ and *R*_p_^–^ reduce to the general form
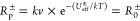
4where *R*_0_^±^ represents the theoretical translocation rate without an applied transmembrane potential (i.e., 0 mV).

Thus
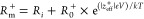
5
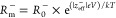
6

If the same barrier potential, *U*_eff_^±^, accounts for interactions with the pore at both positive and negative potentials, this mathematical description implies that data should converge to the same value, *R*_0_^±^, and that *R*_m_^+^ ≠ *R*_m_^–^ at zero applied voltage, assuming that *R*_*i*_ is sufficiently large. Alternatively, a change in the effective barrier energy between the two polarities, which could be created by conformational rearrangements in the amino acid residues of the nanopore, particularly those near the constriction site, creates an offset between *R*_0_^+^ and *R*_0_^–^. Graphically, a change in the barrier energy would shift the vertical positions of the entire positive and negative data sets relative to each other. Additionally, the model states that the magnitude of the effective charge, |*z*_eff_^±^|, is graphically reflected in the slope of the log(translocation rate) vs potential plot.

**Figure 5:**
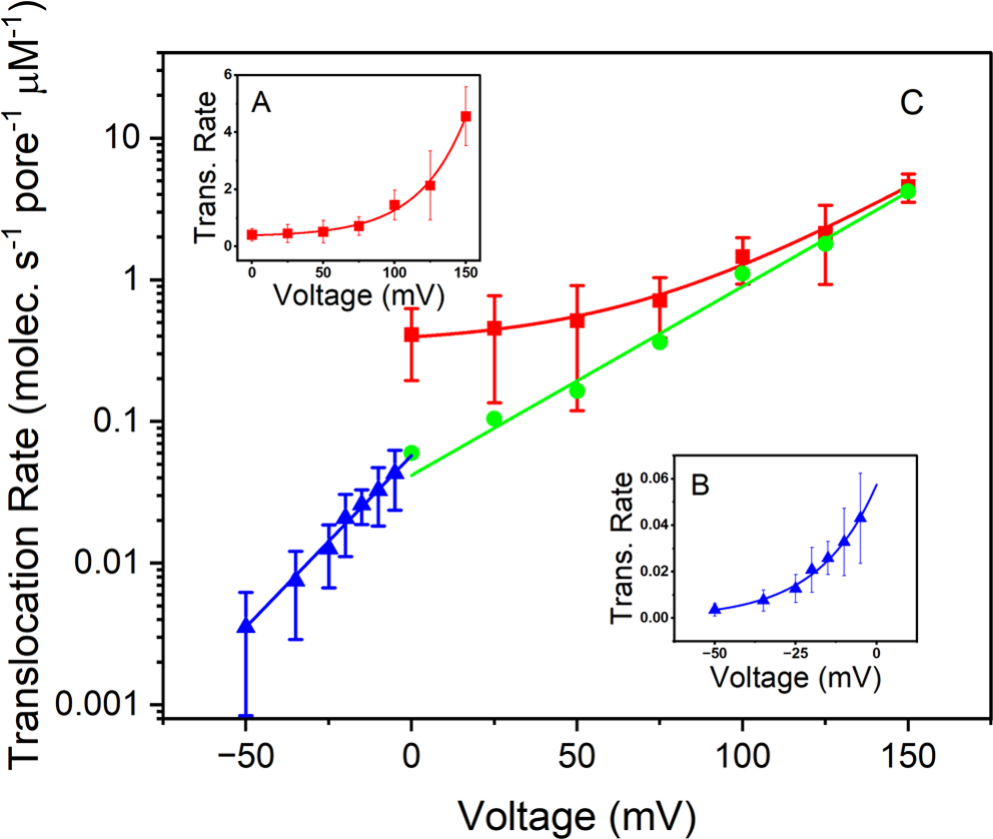
Normalized translocation rate of sCy5a follows an Arrhenius-like potential dependence (see text). For clarity, both linear (A, B insets) and logarithmic (C) scales are shown. Data at positive potentials fit a model that includes a constant offset (red squares). Negative potentials (blue triangles) fit a model without an offset. At all potentials, diffusion contributes to the observed translocation rate. The discontinuity at 0 mV (red to blue) arises from the diffusion of a sCy5a subpopulation that is apparently unaffected by the αHL energy barrier, but is opposed at negative potentials by the reversal of electrophoretic and electroosmotic flow. Subtracting the constant offset rate, *R*_*i*_, from the data at positive voltage (red squares) effectively eliminates the discontinuity (green circles) and reveals the similarity of *R*_0_^±^ values (green and blue @ 0 mV). Determining a value for *R*_0_^±^ permits computation of αHL barrier potentials for both positive and negative potentials (*U*_eff_^±^), which are nearly identical. The effective charge on the dye shifts upon polarity reversal, as indicated by the change in slope at 0 mV (log–linear display). (C) Least-squares analysis of the data yield *U*_eff_^–^ ≈ *U*_eff_^+^ = (7.9–8.1)*kT*, |*z*_eff_^+^| = 0.78, and |*z*_eff_^–^| = 1.4. Error bars (±1 standard deviation) and averages determined from *N* = 5 (blue) or *N* = 3 (red) replicate measurements.

As can be seen in Figure 5, fitting eq 5 to the translocation rate data at positive potentials (with *kT*/*e* = 25.7 mV) results in a good fit (A), yielding |*z*_eff_^+^| = 0.78, *R*_0_^+^ = 0.045 molecules s^–1^ pore^–1^ μM^–1^, and *R*_*i*_ = 0.35 molecules s^–1^ pore^–1^ μM^–1^ (red line). Here, the value of *R*_0_^+^ is established primarily by measurements at positive potentials, where electrophoretic and electroosmotic forces are relatively large and contribute to the observed translocation rate with increasing significance as the potential grows. Previous theoretical studies have demonstrated that diffusive motion dominates electrophoretic and electroosmotic forces throughout a majority of the nanopore lumen at small potentials.^43^ The trend with positive potential that we observe here is consistent with these findings.

Because the potential-independent sCy5a translocation rate (i.e., *R*_*i*_ = 0.35 molecules s^–1^ pore^–1^ μM^–1^) is considerably less than that estimated from the Fickian diffusion of point particles (∼150 molecules s^–1^ pore^–1^ μM^–1^, see the Supporting Information for simulation description and Fickian diffusion rate estimate), we infer that the size of the minority subpopulation unaffected by the αHL barrier is small (i.e., 0.35 molecules s^–1^ pore^–1^ μM^–1^/150 molecules s^–1^ pore^–1^ μM^–1^), ∼0.2% of encounters at 0 mV. The majority of sCy5a molecules (i.e., the remaining ∼99.8% of encounters) interact strongly with the αHL barrier. The relatively small value of *R*_0_^+^ is a consequence of the size of the Arrhenius-like barrier because it prohibits most sCy5a molecules from translocation (i.e., 0.045 molecules s^–1^ pore^–1^ μM^–1^/150 molecules s^–1^ pore^–1^ μM^–1^, 0.03% passage rate at 0 mV). Thus, *R*_0_^+^ represents the transport rate associated with a small fraction of molecules from the strongly interacting (i.e., majority) population that possess enough thermal energy to overcome the barrier and diffusively translocate through the pore without assistance or opposition from applied potential or electroosmotic flow.

As is made especially apparent by the log–linear plot in Figure 5C, the discontinuity at 0 mV between the red and blue data demonstrates that indeed, *R*_m_^+^ ≠ *R*_m_^–^, because *R*_*i*_ is significant. The discontinuity is consistent with the opposition encountered by the reversal of electrophoretic and electroosmotic flow directions. All negative potentials further reduce the transport rate, which is apparently driven by concentration-gradient diffusion. The change at 0 mV is consistent with both the polarity of the net charge on the dye and previous computational studies of the complex electrophoretic and electroosmotic flow directions within the nanoconfined spaces of αHL.^43^ At negative polarity, only those diffusing sCy5a molecules with high enough thermal energy can overcome the combined opposition to translocation imposed by the nanopore barrier, electrophoretic forces, and electoosmotic flow. Furthermore, at increasingly more negative potentials, the fraction of diffusing molecules from the thermal energy distribution with enough energy to overcome the opposition drops exponentially.

The potential-independent term *R*_*i*_ is necessary to fit data at positive potential (red) and is a major contributing factor to the observed translocation rate at small positive potentials. Its contribution is further underscored by the green data set in (C), which is computed as *R*_m_^+^ – *R*_*i*_. As can be seen, removal of *R*_*i*_ makes the positive and negative data sets effectively continuous (i.e., the point at 0 mV is consistent with both sets). The potential-independent term is not, however, necessary to explain trends in the data at negative potentials (B, blue), where only modeling an Arrhenius-like energy barrier is required. Apparently, the concentration gradient drives translocation at negative potentials, but only for an exceedingly small fraction of molecules that most likely approach the barrier with a favorable orientation, proper conformation, and the highest thermal energy.

Similarly, fitting eq 6 to the translocation rate data at negative potentials matches the data well (B) and yields |*z*_eff_^–^| = 1.4, and *R*_0_^–^ = 0.058 molecules s^–1^ pore^–1^ μM^–1^. Thus, similar values are determined for both *R*_0_^+^ and *R*_0_^–^, which indicates that the barrier potential remains nearly constant upon the switch in polarity. This similarity implies that the energy barrier within the nanopore remains constant, and there is little impact from possible conformational changes in the amino acid residues. More significantly, the slope alteration at 0 mV reveals a shift in the effective charge of the dye, which amplifies the retardation of diffusive translocation from the majority population as the potential grows increasingly more negative. Counterion screening from the ionic shell surrounding the dye molecule plays a role in establishing the effective charge extracted from the fit, which remains below 2 for both positive and negative potentials. Thus, the values determined for |*z*_eff_^+^| and |*z*_eff_^–^| are both consistent with the magnitude of charge inherent to the dye. The difference in |*z*_eff_| upon the polarity switch can be interpreted in multiple ways, including a rearrangement of counterions around the dye or a change in the orientation and translocation mechanism of the dye as it diffuses past the barrier at negative potentials. It is also possible that electroosmotic flow plays a significant role in establishing the computed value for both |*z*_eff_^+^| and |*z*_eff_^–^|. Theoretical work indicates that electroosmotic and electrophoretic forces are of similar magnitude within the αHL channel.^43^ However, because the Arrhenius-like model can not account for electroosmotic flow separately, values for |*z*_eff_| should be interpreted accordingly.

To approximate the magnitude of the barrier energy at both positive and negative potentials, we used a Fickian diffusion rate generated by a Brownian dynamics simulation of point particles colliding with the αHL constriction site (see the Supporting Information for details). In the absence of any applied field, the simulation produces an estimate of *v* = 150 collisions s^–1^ pore^–1^ μM^–1^. This is considerably higher than either extracted *R*_0_^±^ value. The difference between simulation and experiment is ascribed to the magnitude of the effective energy barrier, which limits passage to only a small fraction of collisional encounters. Assuming *k* = 1 and *v* = 150, we find that *U*_eff_^+^ = 8.1 *kT* and *U*_eff_^–^ = 7.9 *kT* using both values *R*_0_^±^, through eq 4. Interestingly, both values for the size of the barrier are consistent with the barrier energy reported by others for the translocation of single-stranded polynucleotides through the αHL nanopore (i.e., ∼8 *kT*).^57^

## Conclusions

In summary, we have shown that simultaneous single-molecule fluorescence and single-molecule electrical recordings in a closed aqueous microwell are capable of uncovering nanopore translocation dynamics that have been historically invisible. The findings help dispel commonly held misperceptions that equate molecular translocation with detectable electrical events. Electrical detection becomes possible only when a significant chemical interaction occurs between the translocator and the inner walls of the nanopore. This interaction must be long enough to slow the diffusive movement of the translocator and permit the displacement of a significant quantity of moving charge. Evidently, this kind of interaction is not present for sCy5a despite its charge and size.

More generally, our results demonstrate a direct way to confirm, or refute, the hypothesis of silent passage in ion channels and nanopores. This prospect opens new experimental possibilities relevant to numerous nanopore systems. Although silent translocations have been indirectly described in the nanopore literature in the past, to the best of our knowledge, this study represents the first positive confirmation and characterization of the phenomenon for molecular entities.

Because inherent shot noise from the photon detector can be effectively discriminated, the minimum number of translocated sCy5a molecules that can be detected is background-limited. That is, the smallest translocation rate that can be measured in the microwell is constrained only by a combination of the duration of dye accumulation, the number of nanopores in the bilayer (assuming independent behavior), the magnitude of the concentration gradient, and the subsequent integration time of the optical readout. This permits quantification of exceedingly small translocation rates (i.e., < 0.003 molecules s^–1^ pore^–1^ μM^–1^), as well as translocation events that occur frequently but with electrical silence. Both silent and nonsilent types of passage are important for the general understanding of biological signal transduction and the future development of nanopore-based sensors.

Finally, the translocation rates of sCy5a through αHL follow straightforward trends as a function of dye concentration, accumulation time, and number of nanopores in the membrane. The translocation of sCy5a is a relatively rare event (∼1 in 10^2^–10^3^ encounters with the constriction site, depending on the applied positive potential), most likely due to the relative size of the molecule compared to the constriction region of the nanopore. However, an applied field is not necessary to cause dye passage through the pore. Passive translocation is likely assisted by structural features of the dye that allow the compound to migrate through the lumen without a barrier interaction. For αHL, the relatively small translocation rate at all potentials also underscores the importance of employing a single-molecule fluorescence technique that possesses exquisite sensitivity. For the system studied here, applied fields with a negative polarity reduce the sCy5a translocation rate in an exponential fashion so that translocation is exceedingly rare (i.e., 1 in 10^4^–10^5^ encounters with the constriction site). Characterization of this trend is greatly assisted by an ultrasensitive fluorescence detection method.

## Supporting Information Available

Additional experimental details and materials and methods, including photographs of experimental setup (PDF)
