# Supplementary material for: Confirming Silent Translocation through Nanopores with Simultaneous Single-Molecule Fluorescence and Single-Channel Electrical Recordings
Source: Anal Chem. 2023 Nov 22;95(49):18020–8. doi: 10.1021/acs.analchem.3c02329 (PMC10719886; doi:10.1021/acs.analchem.3c02329)
Supplement: Supplementary file 1 — ac3c02329_si_001.pdf [file ac3c02329_si_001.pdf]

Supporting Information

Confirming Silent Translocation through Nanopores with Simultaneous Single-Molecule Fluorescence and Single-Channel Electrical Recordings

Daniel L. Burden,[1*] Joshua J. Meyer,[1] Richard D. Michael,[1] Sophie C. Anderson,[1] Hannah M. Burden,[1] Sophia M. Peña,[1] Kristin Joy Leong-Fern,[1] Lily Anne Van Ye,[1] Elizabeth C. Meyer,[1] Lisa M. Keranen-Burden[1]

[1] Wheaton College Chemistry Department
*Corresponding author

Table of Content

**Apparatus**	**S3**
**Synchronized Electrical and Optical Data Acquisition**	**S4**
**Membrane Formation, Characterization, and Electrical Monitoring**	**S4**
**Nanopore Addition**	**S4**
**Nanopore Counting, Current Measurement, and Microelectrodes**	**S4**
**Fluid Exchange**	**S5**
**Photobleaching**	**S5**
**Bilayer Translocation Control Measurements**	**S6**
**Detection Volume Size Characterization**	**S6**
**Microwell Calibration and sCy5a Detection Limits**	**S7**
**Computer Simulations**	**S8**

Apparatus

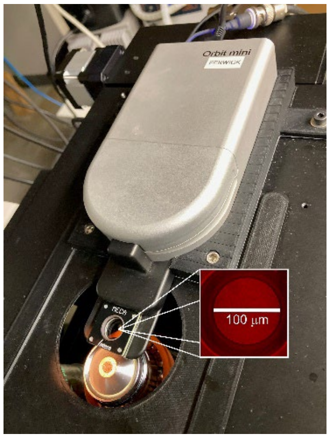

Fig. S1. *Orbit Mini with MECAopto-inv chip. (Inset) Magnified microwell with Ag/AgCl ring electrode. Outer diam. of the microwell is 150 µm with a 100 µm transparent window.*

Electrical measurements were performed using an Orbit Mini Bilayer Workstation (Nanion, Inc.) and a MECAopto-inv chip. The MECAopto-inv chip possesses 4 simultaneously accessible microwells, each with a ring-shaped Ag/AgCl electrode deposited on a thin coverglass (#1.5, 170 µm thick). Microwells are created by photolithograpy in a 25 µm thick SU-8 polymer layer applied on top of the coverglass. The volume of each 150-µm diam. microwell was 440 pL. Microwells were located in the bottom of a single larger well (~0.4 mL) that contained a ground Ag/AgCl electrode. A custom-designed gravity-fed perfusion system was utilized to rapidly perform solution exchange within the chamber. Single-channel current and microwell capacitance recordings were acquired with Elements Data Reader 3 software (EDR3).

The Orbit Mini and the MECAopto-inv chip were mounted on a Nikon Ti-E inverted microscope to perform simultaneous electrical and optical measurements. A motorized stage on the Ti-E allows a single specific microwell to be optically interrogated on demand.

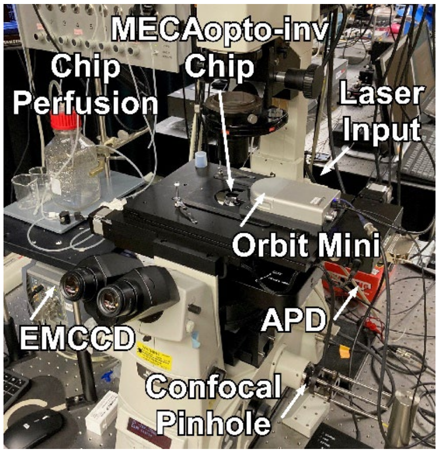

Fig. S2. *Configuration for simultaneous single-molecule fluorescence and single-molecule electrical recording.*

The Ti-E was configured for confocal operation using a collimated 640 nm laser that entered through the rear port of the microscope. The diameter of the beam was adjusted to allow precise filling of the objective back aperture. A dichroic mirror (645 nm) and emission filter (655 nm) were used to direct the beam and isolate fluorescence photons. A Nikon 100x, Plan Apo TIRF, N.A.=1.49 focused the laser to a near diffraction-limited spot, as permitted by the extent of back aperture filling. For all experiments reported herein, the objective was underfilled to create a large volume of detection. A fiber optic pinhole (100 µm diam.) located in an output port of the microscope was positioned in the conjugate focal plane to collect and deliver fluorescence photons to a fiber optically coupled avalanche photo diode (APD) module (SPCM-AQHR, Excelitas). Photocounts from the APD were registered and continually recorded using a multi-channel scalar (MCS, EG&G) in time bins of 1 ms duration. An EMCCD camera (Andor, iXon) was mounted in another output port of the microscope to provide imaging needed for positioning of the microwell.

Synchronized Electrical and Optical Data Acquisition

The TTL digital output connector from the Orbit Mini provided access to a trigger pulse which was used to synchronize continuous optical and current recordings using Protocol 5 (voltage steps of variable length) within the EDR3 software. The software was configured to generate an output timing pulse every 10 seconds. The TTL pulse was split, with one signal sent to the MCS input trigger and another signal sent to a custom-written synchronization program (MasterSynch) created in LabVIEW. Upon receipt of a trigger pulse, the MCS initiated a 9.3 s recording of the APD output and transferred these data to disk upon completion of the recording. The trigger pulse also prompted MasterSych to record the time of the computer operating-system clock, which was synchronized with the MCS and EDR3 system clocks. Thus, the MasterSynch program produced a record of clock times registered in 10-s intervals that allowed each MCS file (optical record) and the EDR3 file (electrical record) to be precisely correlated.

Membrane Formation, Characterization, and Electrical Monitoring

Lipid bilayer membranes were formed using traditional painting techniques.[1] Bilayer capacitance was monitored over time to determine when the membranes were stable and ready for nanopore addition. We used diphytanoyl phosphatidyl choline (DPhPC, Avanti Polar Lipids) in octane (50 mg/mL), which was spread across the four microwell apertures with a bubble manipulated via micropipette tip. The ERD3 software allowed application of a triangle wave to the bilayer. The capacitance of fully sealed and insulating bilayers was determined from the amplitude of the square waves (Fig S3), which allowed periodic computation of the bilayer capacitance. Typically, we sought bilayers that were stable at > 50 pF for 3-5 mins before continuing to the nanopore addition step.

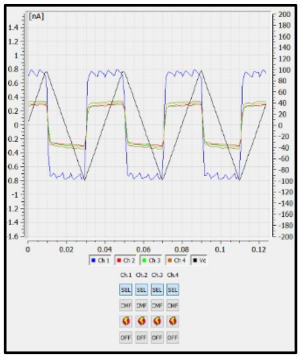

Fig. S3. *Capacitance monitoring of 4 MECAopto-inv microwells with one membrane (blue) that has thinned to create a square wave amplitude that corresponds to a bilayer capacitance of >50 pF.*

Nanopore Addition

Alpha-hemolysin (αHL) was purchased from Sigma-Aldrich, or was produced in-house, using procedures published previously.[2,3] Once a stable bilayer was established, small aliquots of protein monomers (~5 μg/mL) were added to the top chamber of the MECA chip, allowed to bind to the membrane and self-assemble, until individual nanopore insertions were observed. We performed experiments with 1-150 open nanopores inserted in the membrane.

Nanopore Counting, Current Measurement, and Microelectrodes

In order to determine the number of open nanopores in the membrane over the course of an experiment, the average current for a typical open nanopore was determined at each potential. This was established by performing multiple single-nanopore insertion experiments and computing the average of the batch. For experiments with < 40 nanopores, a stepwise increase in current coincided with each nanopore insertion, allowing the total number of nanopores to be directly counted. This number could also be double-checked by dividing the total equilibrium transmembrane current (e.g., after insertions ceased) by the average determined from individual insertion measurements. For experiments with > 40 channels, individual insertions became too difficult to directly count. In this case, the total number of nanopores

was determined by dividing the final equilibrium current by the average current determined from individual nanopore measurements.

The number of open nanopores was also time-averaged over the dye accumulation period to account for random channel losses, or gains, occasional long-lived gating events, or channels with deviant conductance behavior. Channel instability (i.e., increased gating frequency) was particularly noted at the highest positive potential (i.e., 150 mV). To acquire an accurate time-averaged number of open nanopores, the transmembrane current was averaged over the duration of the applied potential and divided by the average nanopore current determined from individual measurements.

To combat bias introduced by electrode drift, the offset current at 0 mV was determined at frequent intervals. The offset was then factored into the total transmembrane current measured at a non-zero potential, which allowed a more accurate count of the total number of open nanopores. Microelectrode polarization, drift, and depletion was of concern for all experiments, but especially those measurements performed with a large numbers of channels (i.e., > 40), a long accumulation time (>500 s), or a large potential (i.e., 150 mV). Periodically, electrode polarization, or Ag/AgCl depletion, deleteriously interfered with measurements and data had to be discarded, along with the chip in which the measurements were performed.

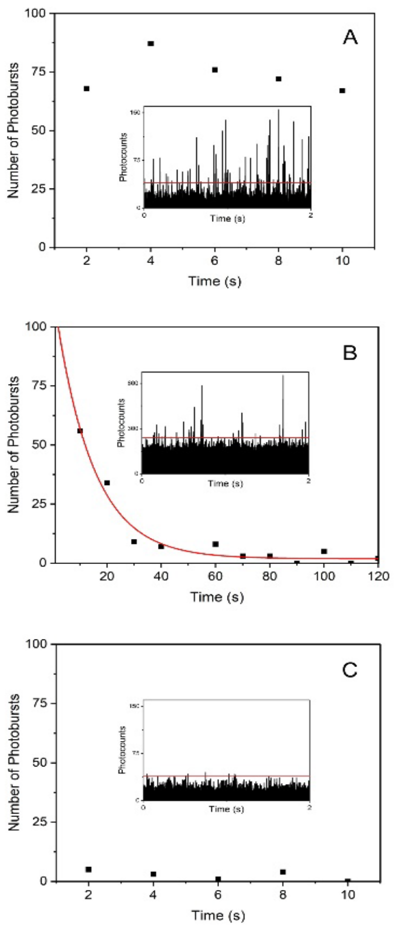

Fig. S4. Optical "zeroing" by photobleaching within a bilayer-capped microwell. Before (A), during (B), and after (C) the photobleach.

Fluid Exchange

A perfusion system was configured to wash out the chamber of the MECAopto-inv chip after successful nanopore addition and to deliver sCy5a dye to the membrane. Following each accumulation period in the microwell, dye was removed from the upper chamber using buffer perfusion. All single-molecule fluorescence measurements in the microwell below the membrane were performed under continuous buffer perfusion to ensure that sCy5a dye was removed from the top chamber during the measurement of translocated dye and keep the fluorescent background low.

Photobleaching

Prior to each accumulation period and translocated dye measurement, microwells were effectively "zeroed" by photobleaching. This was accomplished by increasing the laser power from a nominal reading level (~50-200 μW), to a much higher bleaching level (3 mW), which was maintained for many seconds. High laser power readily photobleached sCy5a, reducing the photoburst count rate to background levels. Fig. S4 shows an example of the microwell bleaching process. Dye molecules residing in the microwell from a previous trial are shown in (A) using 60 μW illumination. The photoburst count rate above the threshold level (inset, red line) is high and does not decrease significantly over the recorded 10-s time interval. Increasing the laser power to 3 mW (B, inset) dramatically enhances scattering signal, which generates a high constant

offset and necessitates a new threshold level to be established, computed at 4 standard deviations above the average background. Typically, 80-100 s of illumination is required to reduce the photoburst count rate to background levels and ready the microwell for a new translocation measurement (B). When the laser power is returned to the reading level, scattering-related signals are reduced (C, inset) and all dye photobursts have been permanently eliminated (C).

Bilayer Translocation Control Measurements

To ensure that DPhPc bilayers were impermeable to sCy5a, we tested for translocation in bilayers without adding αHL. Only bilayers > 50 pF were tested. Experiments were performed with 10 μM dye in the *cis* compartment and a range of applied positive potentials (50-150 mV). After 300 s of applied voltage, we probed the *trans* compartment for the presence of dye. In all experiments, a negligible (i.e., background) number of photobursts (i.e., translocated dye molecules) were observed.

Detection Volume Size Characterization

In order to optimize the detection of translocated dye in the 440 pL microwell of the MECAopto-inv chip, the dimensions of the confocal detection volume (DV) were measured. Using a combination of an underfilled objective and a moderately sized pinhole (relative to the magnification of the objective), we created a DV that was large relative to the diffraction limit. A large DV increases the probability of detecting single molecules in the microwell following nanopore translocation. Individual dye molecules trapped in the microwell produce photobursts as they diffuse in and out of the DV. Moderate-to-low laser powers (50-200 μW) permit translocated dye molecules to enter, exit, and re-enter the DV multiple times before eventually photobleaching. On average, a single trapped dye molecule diffusing within the microwell ($D \approx 3 \times 10^{-6}$ cm^2/s) transits the vertical dimension of the microwell (i.e., ~25 μm) every 0.52 s and the diameter of the microwell (i.e., 150 μm) every 19 s. Counting a single translocated dye molecule multiple times maximizes sensitivity, so that the smallest possible number of molecular translocations can be reliably measured. Although the DV was adjusted for relatively large dimensions, the height was kept small enough to fit inside the 25 μm depth of the microwell. All photoburst acquisition measurements were performed with the beam waist of the DV positioned in the 3D center of the microwell.

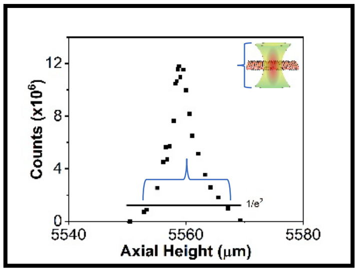

Fig. S5. *Confocal detection volume height determined by an axial scan through a bilayer containing fluorescently labeled lipid. The 1/e^2 height is 12.7 μm.*

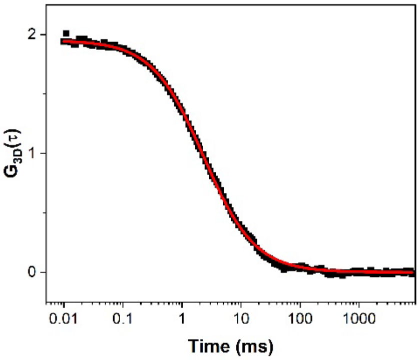

Fig. S6. *The fitted autocorrelation of sCy5a molecules is used to determine <N> and τ_d.*

The geometry of the DV is modeled as a cylinder with radius (ω_1) and half-height (ω_2). We determined the height of the DV by scanning the laser in the axial direction through a fluorescently labeled lipid bilayer (Fig. S5). Because the lipid bilayer is comparatively thin (~5 nm), the resulting intensity peak provided the full height of the cylinder ($2 * \omega_2 = 12.7$ μm). We define boundaries in both axial and radial directions using $1/e^2$ intensity criteria.

The radial dimension of the DV was determined using fluorescence correlation spectroscopy (FCS) and calibration with sCy5a dye standards. First, the total volume was determined by calibrating with a series of sCy5a concentration standards, ranging from 0-50 pM. A single-component correlation curve contains concentration-related information in its amplitude and diffusion-related information in its width. Autocorrelation curves produced from the fluorescence intensity vs. time trace (Fig. S6) were fit with the equation, $G_{3D}(\tau) = \frac{[\langle F(t)\rangle]^2}{[\langle F(t)\rangle + \langle B(t)\rangle]^2}\frac{0.35}{\langle N\rangle}(1+\frac{\tau}{\tau_D})^{-1}(1+\frac{\tau}{K^2\tau_D})^{-1/2}$, where $<F(t)>$ is the measured average fluorescence signal and $<B(t)>$ is the measured average background, $<N>$ is the average number of molecules that reside inside the detection volume, τ_D is the average crossing time for a dye molecule and $K = \omega_2/\omega_1$. An initial estimate of $K = 13$ yields fitted values for both $<N>$ and τ_D. Determination of $<N>$ as a function of standard concentration (in molecules/L) produces a linear relationship with a slope that reveals the size of the DV in L (Fig. S7).

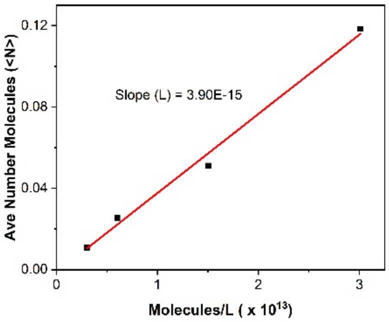

Fig. S7. Values of <N> plotted as a function of standard concentration give a slope equal to the detection volume of confocal probe region.

The DV established for our optical configuration was 3.9E-15 L. This enables the radial dimension (ω_1) to be estimated from the formula for the volume of a cylinder ($V=2\pi\ \omega_2\omega_1^2$). Employing the direct measurement of $2\omega_2 = 12.7$ μm produces an ω_1 value of 0.312 μm, which also gives a refined estimate of $K = 20$.

Microwell Calibration and sCy5a Detection Limits

The number of translocated dye molecules is determined by counting photobursts as a function of time, using threshold criteria established at > 99.99% confidence for Gaussian noise (i.e., 4 standard deviations above the average background). To determine concentration, microwells were calibrated using standard dye solutions. A typical calibration curve is shown in Fig. S8. Photoburst count rates maintain linearity as a function of concentration within the range of ~3-100 pM. Non-linearities begin to appear outside of this range. At concentrations below 3 pM, positive deviations occur due to trace-level background contamination. Above 100 pM, negative deviations begin to occur due to the overlap of photobursts, which are caused by more than one dye molecule residing in the DV simultaneously. All dye translocation experiments performed at a positive applied potential were measured in the linear region of the calibration curve. Translocation measurements performed at negative potentials extended into the low-concentration region of the calibration curve, approaching the minimum detectable quantity for a 9.3-s integration period.

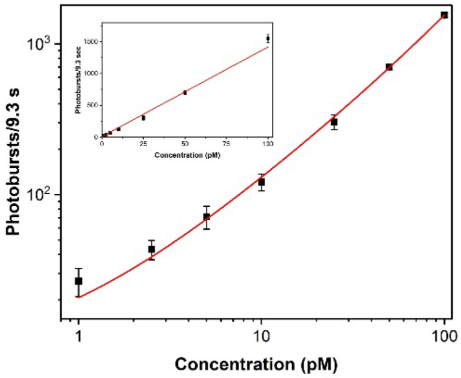

Fig. S8. Typical microwell calibration derived from sCy5a photoburst count rates in logarithmic and (inset) linear format.

In order to establish the minimum accumulation time needed for a given experiment, it is important to compute the detection limit and arrive at the smallest number of dye molecules that can be reliably identified in a microwell. For this, blanks were evaluated using a "read" laser power (50-200 μW) to quantify the average number of spurious photobursts in a 9.3 s window. The detection-limit concentration was

computed at 99.9% confidence (i.e., 3 standard deviations above average background level) and was determined to be ~0.3 pM, which represents ~10^2 molecules in the 440 pL volume of the MECAopto-inv microwell.

Computer Simulations

In order to determine a theoretical estimate of the collision frequency in the absence of any applied voltage, ν, we constructed a random walk computer simulation to mimic Brownian motion inside and around the αHL nanopore. Diffusion was modeled as point particles colliding with an absorbing disk. We presumed the absorber to represent the constriction site within the αHL lumen, assuming that penetration of sCy5a beyond this location resulted in irreversible translocation events. The algorithm we employed was similar to that published previously[4] and is briefly summarized here.

First, a point particle was positioned at a randomly chosen location within a 20-nm simulated cube and the total number of diffusive steps to be executed for a user-specified concentration and time period was calculated. The particle was then moved at random through the three-dimensional space by single integer steps in one of 6 cardinal directions. For computational efficiency, the simulation operated on a single particle at a time, moving it through space for a pre-determined number of steps, or until the particle collided with the absorbing disk. When a particle contacted the disk, it was removed from the simulation and its unused steps were allotted to another particle, which was then repositioned within the cube at random. In this way, loss of particles to the sink did not affect the average simulated concentration. This approximates reality because bulk dye concentration is unaffected by translocation through the nanopore. Periodic boundary conditions were enforced when the particle collided with one of the 6 walls of the cube. The simulation was terminated once the maximum number of allotted steps for a given concentration and specified simulation time were completed. The simulation was designed to scan through a range of absorbing disk radii and the number of collisions with the disk for each radius size during the simulated time period was totaled. In addition, the average particle density was determined at each location within the cube, which is displayed as a function of blue-to-white color gradient in the 2D cross-section shown in Fig. S9.

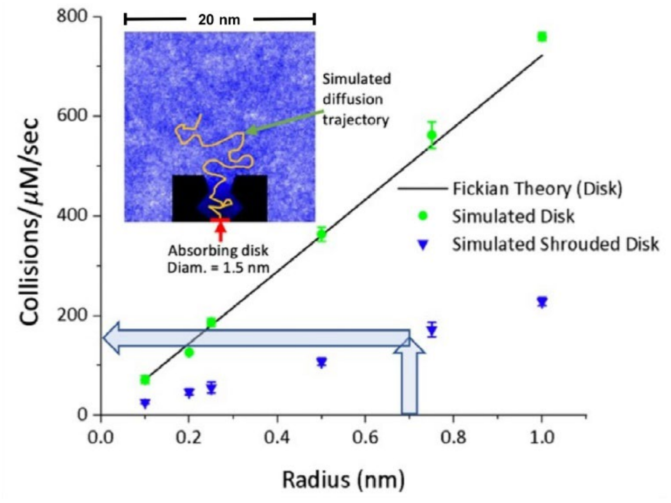

Fig. S9. *Estimation of the collision frequency with the αHL constriction site (ν). Diffusion of point particles to an absorbing disk simulated in a 3D cube (cross section shown in inset). Collision rate for unobstructed diffusion to an absorbing disk (green circles). Fickian theory for D=3 x 10^{-6} cm^2/s, C_o=1 μM, and r (black line). The αHL cap (black solid profile) obstructs access to the simulated constriction site (i.e., absorbing disk). The specific constriction site radius determined from the αHL crystal structure (i.e., 0.7 nm) allows the collision frequency (blue triangles) to be estimated ($\nu \cong 150$ collisions s^{-1} $pore^{-1}$ μM^{-1}).*

Fickian theory for diffusion to a disk-like absorber predicts a linear relationship between disk radius and collision frequency (ν):[5]

$$\nu = 4 * D * r * Co$$

where D is the diffusion coefficient, r is the radius of the disk, and C_o is the concentration. We used this theory to verify simulation accuracy (Fig. S9) by comparing the computed collision frequency (green circles) to the predicted Fickian theory (black line). Both were in close agreement.

To obtain a more realistic measure of the collision frequency with the constriction site inside αHL, a 3D shroud, approximating the size and contour of the αHL cap (black), was positioned around the absorbing disk (red). The occluding shroud reflects particles that contact its surface. As expected, the shroud obstructed access to the disk, significantly lowering the overall collision rate. However, the collision rate remained linear as a function r, D and C_o. Using the radius of the constriction site taken from the αHL crystal structure (0.7 nm),[6] as well as the approximate diffusion constant for sCy5a in aqueous solution,[7,8] the collision frequency in the absence of applied potential was estimated to be $\nu \cong 150$ collisions/μM/s.

This computed collision frequency is significantly higher than the measured translocation rate for sCy5a in the absence of applied potential and diffusive transport (~0.045 translocations/μM/s). The difference is explained by an effective Arrhenius-like energy barrier to translocation. The theoretical collision frequency computed here most likely represents an upper limit, because real particles with a finite structural volume and an associated solvation shell reduce the cross-sectional opening at the mouth of the nanopore, effectively reducing the probability of the particle's entry. A high estimate for ν corresponds to an artificially high estimate for the barrier energy. Nonetheless, the barrier energy we computed at zero applied voltage for positive and negative data sets (~7.9-8.1 kT) compares favorably with that found by others for DNA translocation (~8 kT).[9] Furthermore, our previous simulations,[4] in both the presence and absence of the small experimental potentials used in this work (-50 mV to +150 mV), suggest that point particles provide a reasonable approximation of the collision frequency with the constriction region for all reasonable radii of the nanopore.

REFERENCES

(1) Mueller, P.; Rudin, D. O.; Tien, H. T.; Wescott, W. C. Reconstitution of Cell Membrane Structure in Vitro and Its Transformation into an Excitable System. *Nature* **1962**, *194*, 979–980. https://doi.org/10.1038/194979a0.

(2) Burden, D. L.; Kim, D.; Cheng, W.; Lawler, E. C.; Burden, L. M. K. Mechanically Enhancing Planar Lipid Bilayers with a Minimal Actin Cortex. *Langmuir* **2018**, *34* (37), 10847–10855. https://doi.org/10.1021/acs.langmuir.8b01847.

(3) Chandler, E. L.; Smith, A. L.; Burden, L. M.; Kasianowicz, J. J.; Burden, D. L. Membrane Surface Dynamics of DNA-Threaded Nanopores Revealed by Simultaneous Single-Molecule Optical and Ensemble Electrical Recording. *Langmuir* **2004**, *20* (3), 898–905. https://doi.org/10.1021/la035728i.

(4) Pederson, E. D.; Barbalas, J.; Drown, B. S.; Culbertson, M. J.; Keranen Burden, L. M.; Kasianowicz, J. J.; Burden, D. L. Proximal Capture Dynamics for a Single Biological Nanopore Sensor. *J. Phys. Chem. B* **2015**, *119* (33), 10448–10455. https://doi.org/10.1021/acs.jpcb.5b04955.

(5) Berg, H., C. *Random Walks in Biology: New Expanded Edition*, Rev.; Princeton University Press: 41 William St., Princeton, NJ 08540, 1993.

(6) Song, L.; Hobaugh, M. R.; Shustak, C.; Cheley, S.; Bayley, H.; Gouaux, J. E. Structure of Staphylococcal Alpha-Hemolysin, a Heptameric Transmembrane Pore. *Science* **1996**, *274* (5294), 1859–1866. https://doi.org/10.1126/science.274.5294.1859.

(7) Bark, N.; Földes-Papp, Z.; Rigler, R. The Incipient Stage in Thrombin-Induced Fibrin Polymerization Detected by FCS at the Single Molecule Level. *Biochem. Biophys. Res. Commun.* **1999**, *260* (1), 35–41. https://doi.org/10.1006/bbrc.1999.0850.

(8) Mozziconacci, J.; Sandblad, L.; Wachsmuth, M.; Brunner, D.; Karsenti, E. Tubulin Dimers Oligomerize before Their Incorporation into Microtubules. *PLoS ONE* **2008**, *3* (11), e3821. https://doi.org/10.1371/journal.pone.0003821.

(9) Henrickson, S. E.; Misakian, M.; Robertson, B.; Kasianowicz, J. J. Driven DNA Transport into an Asymmetric Nanometer-Scale Pore. *Phys. Rev. Lett.* **2000**, *85* (14), 3057–3060. https://doi.org/10.1103/PhysRevLett.85.3057.
